# Adenosine triphosphate treatment affects strawberry fruit quality by regulating sugar and organic acid metabolism

**DOI:** 10.3389/fnut.2026.1782642

**Published:** 2026-02-18

**Authors:** Jiahui Cai, Xinrong Dai, Huixin Fang, Guozhen Tian

**Affiliations:** College of Food Science and Engineering, Bohai University, Jinzhou, Liaoning, China

**Keywords:** adenosine triphosphate, fruit ripening, organic acid metabolism, strawberries, sugar metabolism

## Abstract

**Introduction:**

Strawberries are characterized by distinctive flavors, sweet taste profiles, and abundant bioactive nutrients. However, they have a tender texture, and are prone to rapid ripening and softening post-harvest, leading to a loss of their commercial quality and marketability. Exogenous adenosine triphosphate (ATP) at appropriate concentrations can activate beneficial extracellular ATP signaling pathways that preserve agricultural product quality and extend shelf life during storage. However, research on the effects of exogenous ATP on strawberries is limited. Therefore, we aimed to analyze the regulatory effects of different concentrations of ATP on strawberry ripening by exploring its effects on sugar and organic acid metabolism in strawberries.

**Methods:**

The effects of different concentrations of ATP on the quality, sugar metabolism, and organic acids of strawberry fruits stored at 4 °C for 15 days were examined.

**Results:**

The results demonstrated that 1 mM ATP effectively maintained fruit color, promoted the accumulation of soluble solids, reduced titratable acidity, and inhibited the decline in pulp firmness in sugar metabolism. Treatment with 1 mM ATP promoted the accumulation of glucose, fructose, and sucrose in strawberries, and enhanced the activities of acid invertase (AI), neutral invertase (NI), sucrose synthase (SS-s), sucrose cleavage enzyme (SS-c), and hexokinase (HK). It upregulated the expression of *FaAI*, *FaSS1*, *FaSPS1*, *FaSPS2*, *FaNI*, and *FaHK3*. In organic acid metabolism, 1 mM ATP treatment promoted the degradation of citrate and malate, and enhanced the activities of phosphoenolpyruvate carboxylase (PEPC) and NADP-dependent malic enzyme (NADP-ME), but reduced the activities of ferredoxin NADP-reductase-linked malate dehydrogenase (NAD-MDH) and citrate synthase (CS) enzymes. It also upregulated the gene expression of *FaPEPC* and *FaNAD-IDH*, and downregulated the expression of *FaCS5*.

**Conclusion:**

Overall, 1 mM ATP treatment maintained strawberry fruit quality by regulating the expression of key genes and enzyme activities involved in sugar and organic acid metabolism in strawberries, thereby extending its shelf life.

## 1 Introduction

Strawberries belong to the berry fruit category, which are characterized by a distinctive sensory flavor and contain several diverse bioactive ingredients (1). During the ripening period, strawberries gradually turn bright red, accompanied by a fragrant aroma and sweet–sour taste (2). However, the fragile tissue and juicy flesh characteristics of strawberries make them highly susceptible to mechanical damage, leading to microbial invasion and consequently limiting their post-harvest storage period, which directly reduces their market acceptance and product value (3, 4). Therefore, it is important to investigate strawberry-ripening regulation technologies for strawberry storage and preservation.

Adenosine triphosphate (ATP) can act as a signaling molecule that mediates a variety of biological reactions. Deficiency of intracellular ATP triggers abnormal physiological responses and shortens shelf life. Conversely, maintaining stable intracellular ATP levels helps delay the senescence of fresh fruits and vegetables during storage and suppress stress-induced damage (5). Exogenous ATP affects the quality of post-harvest fruits and vegetables during storage by regulating extracellular ATP signaling functions (6). Exogenous ATP at appropriate concentrations can activate beneficial extracellular ATP signaling pathways, thereby preserving agricultural product quality and extending shelf life during storage. ATP treatment demonstrated significant positive effects on the shelf life, and quality of various fruits and vegetables, such as banana (6), pear (7), mung bean sprouts (8), longan fruit (9, 10), and *Agaricus bisporus* (11).

The perception of sweetness and sourness in fruits is primarily influenced by the soluble sugar and organic acids (12). Soluble sugars, such as sucrose, glucose, and fructose, play a crucial role in various physiological and morphological processes of plants, primarily through two aspects: providing carbon sources and participating in signaling pathways (13, 14). In addition, they are crucial for respiration and osmoregulation, and provide carbon sources for the biosynthesis of other metabolites (such as amino acids, organic acids, and flavonoids) (13). The homeostasis of soluble sugars is mediated by key enzymes, including acid invertase (AI), neutral invertase (NI), sucrose synthase (SS-s), and sucrose phosphate synthase (SPS).

Homeostasis of soluble sugars is governed by enzymes, including neutral invertase (NI), acid invertase (AI), sucrose phosphate synthase (SPS) and sucrose synthase (SS-s) (15). Exogenous ATP treatment significantly alleviated the decrease in firmness of post-harvest grape berries, reduction in total soluble solids (TSS), and decrease in vitamin C content. It also effectively delays the loss of fruit sugars and the ripening process of fruit quality (16). ATP (0.8 mM) significantly reduces the respiratory intensity and increase TSS and flesh firmness of Nanguo pear fruits. Additionally, ATP treatment enhances the activity of AI, NI, SS-s, and sucrose cleavage enzymes (SS-c). Therefore, ATP helped maintain the quality of Nanguo pear fruit by regulating the activity of sucrose metabolizing enzymes (7).

Variation in the organic acid content of fruits can be attributed to the dynamic balance between their synthesis and degradation processes (17). The tricarboxylic acid cycle is the primary pathway for the synthesis of organic acids, such as malic and citric acid (18). Malic acid accumulation and decomposition are regulated by NAD-malate dehydrogenase (NAD-MDH), NADP malic enzyme (NADP-ME), and phosphoenolpyruvate carboxylase (PEPC) (19). Citrate synthase (CS) is involved in citrate metabolism (20). ATP can effectively inhibit the decrease in TSS, total soluble sugars, and vitamin C content in longan flesh, and limit the increase in titratable acidity (TA) content, thus maintaining the quality and flavor of the longan flesh (21).

Zhang et al. (22) demonstrated that ultrasonic treatment effectively inhibited post-harvest softening of strawberries, preserved fruit firmness, and reduced soluble pectin production, thereby contributing to the maintenance of strawberry texture quality. Wang et al. (23) demonstrated that the combined application of 1-MCP and H_2_S treatment is an effective and potential method for maintaining post-harvest strawberry quality. However, research on the effects of exogenous ATP treatment on strawberries is insufficient. Therefore, we primarily analyzed the regulatory effects of different concentrations of ATP on strawberry ripening by exploring its effects on sugar and organic acid metabolism in strawberries. This study will enhance our understanding of the regulatory methods for strawberry fruit ripening, and provide new insights for strawberry preservation and storage.

## 2 Materials and methods

### 2.1 Plant materials and treatments

Strawberries (*Fragaria × ananassa* Duch. cv. “Benihoppe”) were harvested from a local orchard in Jinzhou, Liaoning Province, China. The fruits were harvested at 70–80% maturity, had a uniform size, and were free from mechanical damage. Approximately 200 fruits were collected and immediately transported to the laboratory in foam boxes and randomly divided into four groups. Three groups were soaked in ATP solutions at concentrations of 0.5, 1, and 5 mM for 10 min, and the control group was soaked in distilled water for 10 min. Subsequently, they were air dried at room temperature (22 °C ± 0.5 °C), packed in polyethylene bags (0.02 mm thick), and stored at 4 °C ± 0.5 °C and 85% relative humidity. Every 3 days during the storage period, the middle section of the strawberry fruit was sampled from each treatment and frozen at −80 °C. Each experiment was repeated three times with biological and technical replicates.

### 2.2 Color values and firmness measurements

The color change of strawberries was determined using a digital colorimeter (CR-20, Konica Minolta, Tokyo, Japan). After calibration with a standard board, the values of L*, a*, and b* were measured at the center of strawberries. L* can effectively assess the color change of strawberries during storage, especially the appearance of brown and dark colors. a* and b* represent the color positions on the red-green axis and yellow-blue axis, respectively. The larger a* value indicates more red components, while the larger b* value denotes more yellow components.

A texture analyzer (TA-XT2i Plus, Ruifen, Shanghai, China) was used to measure the firmness of strawberries with a 2 mm probe diameter and 5 mm pressing distance. Each sample was measured six times.

### 2.3 Determination of TSS and TA

Samples collected from each fruit were pooled and juiced for measurement according to a method described by Saba (24). The TSS values were measured using a portable digital refractometer (PR-10101; Atago, Tokyo, Japan). TA was measured by titrating 10 mL of juice with 0.1 mol L^−1^ NAOH to pH 8.1 and expressed as a percentage of malic acid.

### 2.4 Determination of sugar metabolism and organic acid content

Sugar metabolism and organic acid content measurements were performed according to the manufacturer’s instructions (Enzyme-Linked Biotechnology, Shanghai, China). Glucose (GLC-W96-N1620), fructose (FT-W96-N1620), and sucrose (SC-W96-N1620) contents were quantitatively analyzed using an enzyme immunoassay analyzer (Victor X3, PerkinElmer, MA, USA). Malic (MA-F96-N1620) and citric acid (CA-F96-N1620) contents were quantitatively analyzed using a microplate reader (Evolution 201, Thermo Fisher Scientific, MA, USA), with three biological replicates for each sample.

### 2.5 Extraction and assay of enzymes activity related to sugar metabolism

The frozen samples (4 g) were taken for the extraction of AI, NI, SS-s and SS-c according to a method described by Zhang et al. (25). HK activity was determined using the extraction method proposed by Basson CE (26). A measurement method proposed by Sun et al. (27) was used to determine AI and NI activity, the absorbance was measured at 540 nm. The SS-c activity was examined according to Duan e al. (7). According to a method proposed by Solomakhin et al. (28), SS-c synthetic activity was measured, and the absorbance was measured at 620 nm. HK activity was determined following the experimental protocol established by Doehlert (29), and the absorbance was measured at 340 nm. All the results were expressed as μmol h^−1^ g ^-1^FW.

### 2.6 Extraction and assay of enzymes activity related to organic acid

The frozen samples (1 g) were taken for the extraction of PEPC, CS, NAD-MDH, and NADP-ME, according to a method described by Millaleo (30). Activities of PEPC, NAD-MDH, NADP-ME, and CS were measured and in accordance with a method by Chen et al. (31). The absorbance of the NADP-ME, NAD-MDH, CS, and PEPC reaction systems was measured at 340 nm, the results were expressed as U g^−1^ FW.

### 2.7 Analysis of gene expression

Total RNA was extracted using a Plant RNA Extraction Kit (Waryong, Beijing, China) following the manufacturer’s instructions. An *Evo M-MLV*RT Mix Kit with gDNA Clean (Accurate Biology, Hunan, China) was used for cDNA synthesis. Using the synthesized cDNA as a template, real-time quantitative PCR reactions were performed for *FaAI*, *FaSS1*, *FaSPS1*, *FaSPS2*, *FaNI*, *FaHK3*, *FaPEPC*, *FaNAD-IDH,* and *FaCS5*. Gene-specific primers used are listed in Supplementary Table 1. Amplification was performed using a real-time PCR detection system with a SYBR Green Premix *Pro Taq* HS qPCR Kit (Accurate Biology). The relative gene expression was normalized against the relative value of the *FaActin* and the amplification products were analyzed using the 2^−ΔΔCT^method for melt curve analysis.

### 2.8 Statistical analysis

Each treatment was conducted in triplicate to ensure reliability of the experimental results. Data analysis was performed using SPSS software (version 19.0; IBM, Armonk, NY, USA). Results are presented as mean ± standard deviation. Significance analysis was based on Duncan’s multiple range test, with the significance level set at *p* < 0.05. Charts were drawn using the Prism 8 software (GraphPad Inc., La Jolla, CA, USA).

## 3 Results

### 3.1 Changes in ripening parameters of strawberries during storage

**Figure 1:**
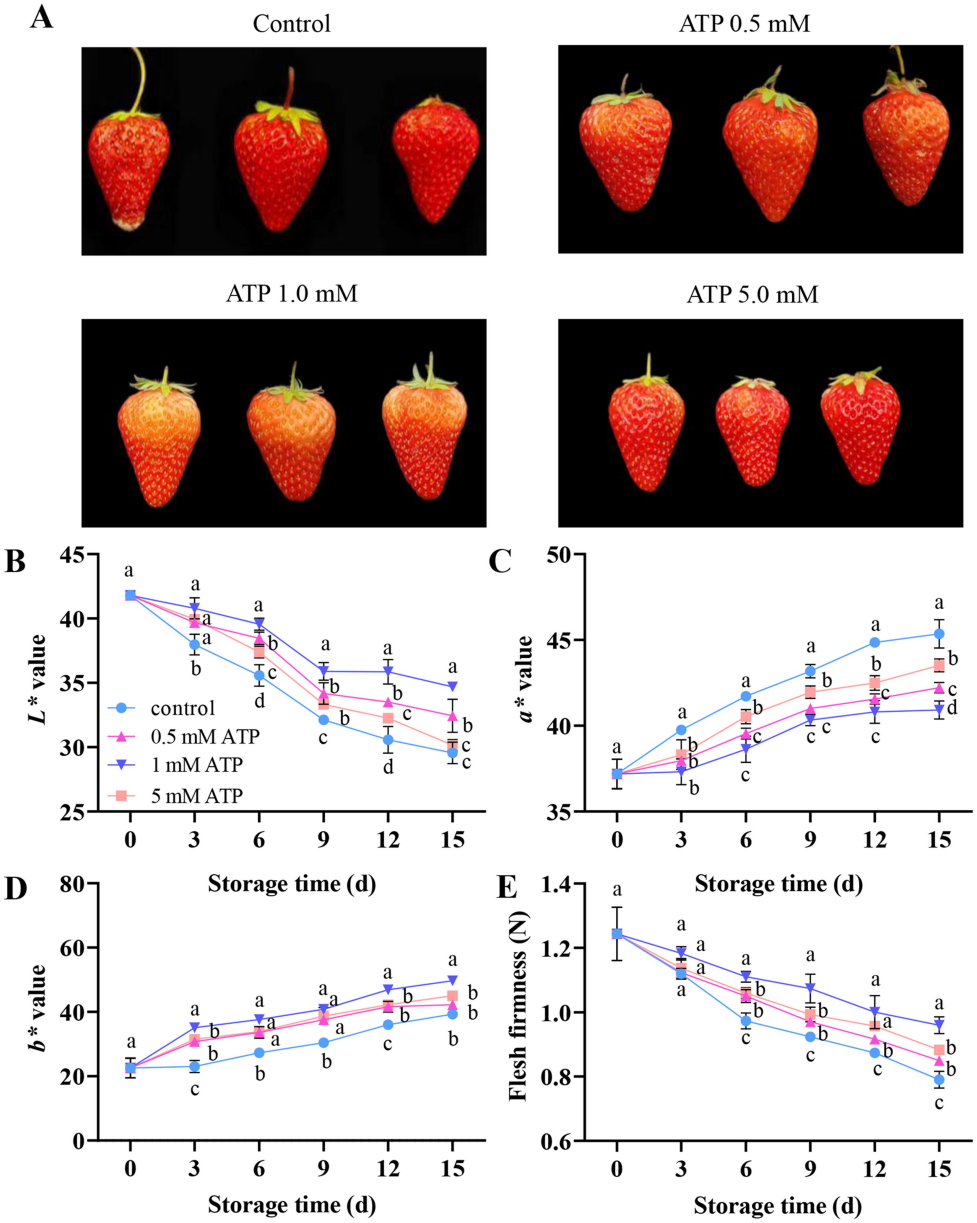
The ripening progress of strawberries stored at 4 °C. **(A)** Image of fruits treated with different concentrations of ATP [control (0 mM ATP), 0.5, 1, and 5 mM] at 15 d after treatment. **(B–D)** Color changes. **(E)** Firmness. Each value is the mean ± standard deviation from at least three strawberries. Different letters indicate significant differences among different treatments (*p* < 0.05). ATP, adenosine triphosphate.

To monitor changes in important ripening characteristics during fruit storage, the color and firmness of the fruits treated with different concentrations of ATP were measured. The untreated strawberries were fully ripened after 15 days of storage, accompanied by some rotting. While ATP treatment significantly delayed the ripening of strawberries, fruits treated with 1 mM ATP did not fully turn red on day 15 (Figure 1A). As shown in Figure 1B, the L* values of both untreated and ATP-treated strawberries during storage showed a downward trend, indicating darkening of the color of the fruit samples. However, through comparative analysis, it was evident that the L* values of strawberries treated with 1 mM ATP were significantly higher than those of untreated samples, exhibiting a more lustrous appearance. As the storage time increased, the value of a* increased, indicating that strawberries gradually turned red and ATP treatment delayed this process (Figure 1C). The value of b* increased after storage and ATP treatment delayed it (Figure 1D). As shown in Figure 1E, with the extension of storage time, firmness showed a decreasing trend. The 1 mM ATP treatment significantly delayed the decline in fruit firmness, indicating that ATP treatment delayed fruit ripening.

### 3.2 Sugar content changes in strawberries

**Figure 2:**
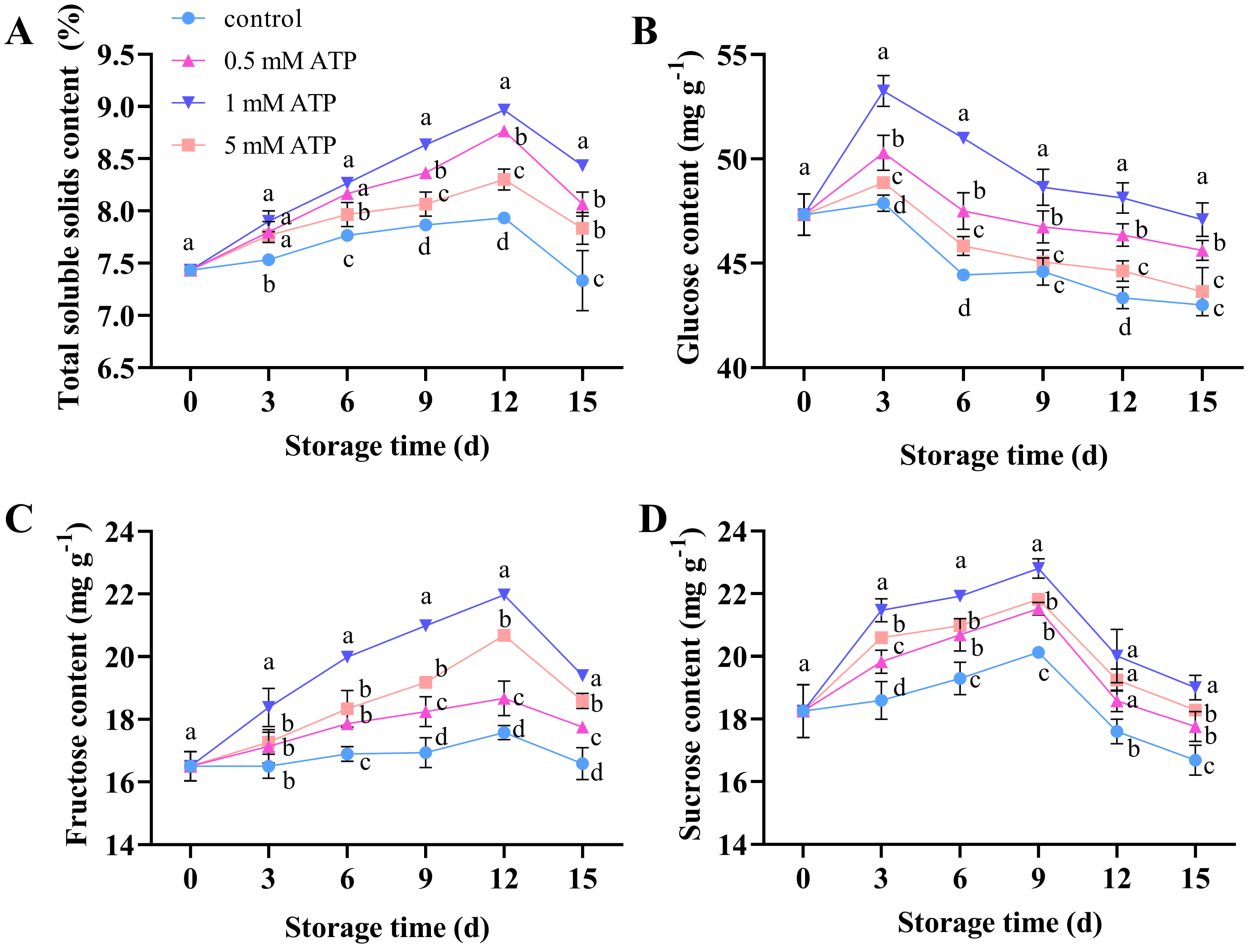
The effect of different concentrations of ATP treatment on the sugar content of strawberry fruit **(A–D)**. **(A)** TSS. **(B)** Glucose content. **(C)** Fructose content. **(D)** Sucrose content. Each value is the mean ± standard deviation from at least three strawberries. Different letters indicate significant differences among different treatments (*p* < 0.05). ATP, adenosine triphosphate; TSS, total soluble solid.

As shown in Figure 2A, with an increase in storage time, the TSS in strawberries showed an upward trend, reaching a peak on day 12 and then decreasing. ATP treatment increased the content of soluble solids in strawberries, among which the 1 mM ATP treatment had the most significant effect. The glucose, fructose, and sucrose contents first increased and then decreased, reaching their peaks on days 2, 12, and 9, respectively, (Figures 2B–D). Different concentrations of ATP significantly increased the glucose, fructose, and sucrose content, especially in the 1 mM ATP treatment. It is speculated that exogenous treatment with 1 mM ATP could significantly enhance the contents of sucrose, fructose, and glucose in strawberry fruits, thereby contributing to an increase in the TSS content.

### 3.3 Organic acid content changes in strawberries

**Figure 3:**
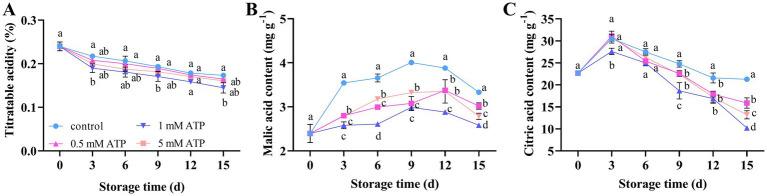
The effect of different concentrations of ATP treatment on the organic acid content of strawberry fruit. **(A)** TA. **(B)** Malic acid content. **(C)** Citric acid content. Each value is the mean ± standard deviation from at least three strawberries. Different letters indicate significant differences among different treatments (*p* < 0.05). ATP, Adenosine triphosphate; TA, titratable acidity.

As shown in Figure 3A, the TA showed a downward trend with prolonged storage. Compared with the control, ATP treatment decreased TA. The malic and citric acid contents first increased and then decreased with increasing storage time, with the peak value of untreated strawberries being achieved on days 9 and 3, respectively. Malate and citrate content in the presence of 1 mM ATP were consistently lower than those in the control (Figures 3B,C). Therefore, exogenous treatment with 1 mM ATP significantly decreased the contents of malic acid and citric acid, thereby contributing to an decrease in the TA content.

### 3.4 Activities of sugar metabolism related enzymes in strawberries

**Figure 4:**
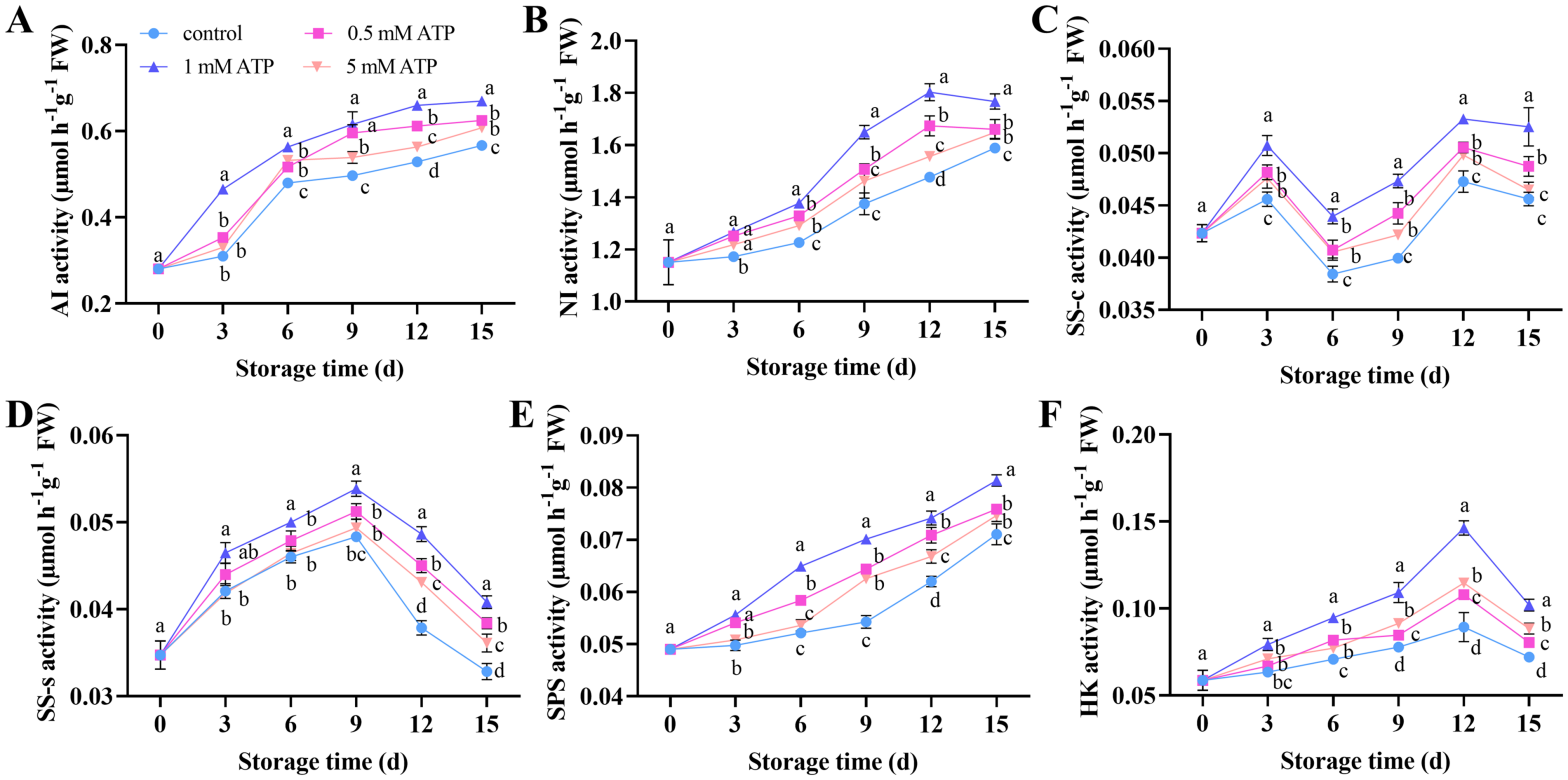
Changes in sugar metabolism related enzyme activities in strawberries. **(A)** AI. **(B)** NI. **(C)** SS-c. **(D)** SS-s. **(E)** SPS. **(F)** HK. Each value is the mean ± standard deviation from at least three strawberries. Different letters indicate significant differences among different treatments (*p* < 0.05). AI, acid invertase; HK, hexokinase; NI, neutral invertase; SS-c, sucrose cleavage enzyme; SS-s, sucrose synthase; SPS, sucrose phosphate synthase.

Sugar is a fundamental substance for fruit growth and development, and serves as a substrate, intermediate reactant, and energy source for various metabolic processes in fruits. The enzyme activities of AI, NI, and SPS showed an upward trend (Figures 4A,B,E). The activities of the SS-s and HK enzymes exhibited a trend of first increasing and then decreasing, with peak values on days 9 and 12, respectively (Figures 4D,F). The SS-c enzyme activity first increased to reach a peak on day 3, followed by a decrease; subsequently, it increased again and reached another peak on day 12, after which a further decrease was observed (Figure 4C). ATP treatment increased the activity of enzymes related to sugar metabolism, among which the 1 mM ATP treatment exhibited the most significant promoting effect.

### 3.5 Activities of organic acid related enzymes in strawberries

**Figure 5:**
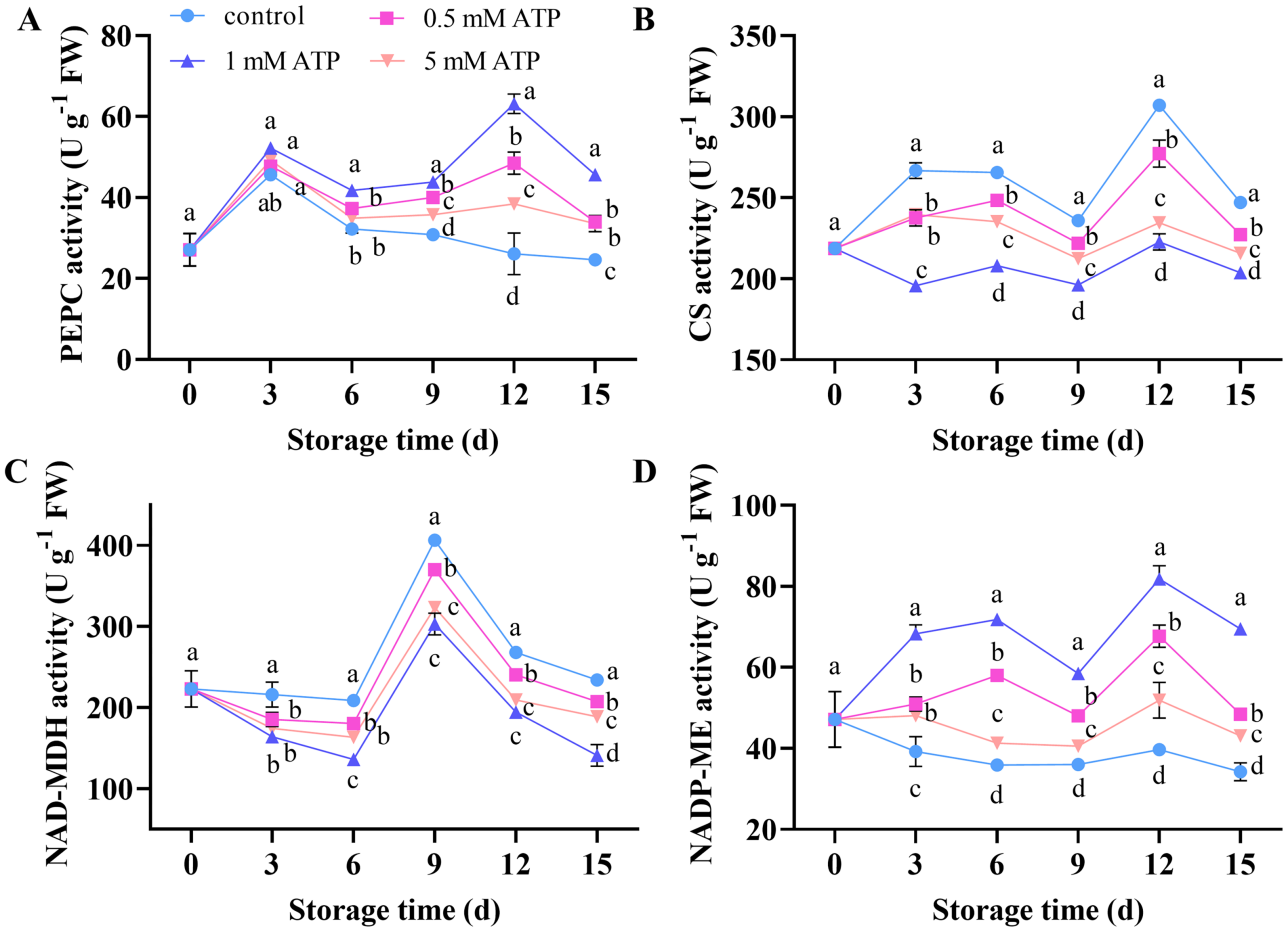
Changes in organic acid related enzyme activities in strawberries. **(A)** PEPC. **(B)** CS. **(C)** NAD-MDH. **(D)** NADP-ME. Each value is the mean ± standard deviation from at least three strawberries. Different letters indicate significant differences among different treatments (*p* < 0.05). CS, Citrate Synthase; NADP-MDH, NADP malate dehydrogenase; NADP-ME, NADP malic enzyme; PEPC, phosphoenolpyruvate carboxylase.

The enzymatic activities of PEPC, CS, and NADP-ME peaked on days 3, 3, and 6, respectively, and then decreased. Subsequently, it increased again, peaked on day 12, and then decreased (Figures 5A,B,D). The NAD-MDH enzyme activity exhibited a trend of first increased, peaked on day 9, and then decreased (Figure 5C). ATP treatment increased the activities of PEPC and NADP-ME, and decreased the activities of CS and NAD-MDH, among which the 1 mM ATP treatment exhibited the most significant promoting effect.

### 3.6 Sugar metabolism related genes involved in strawberries ripening

**Figure 6:**
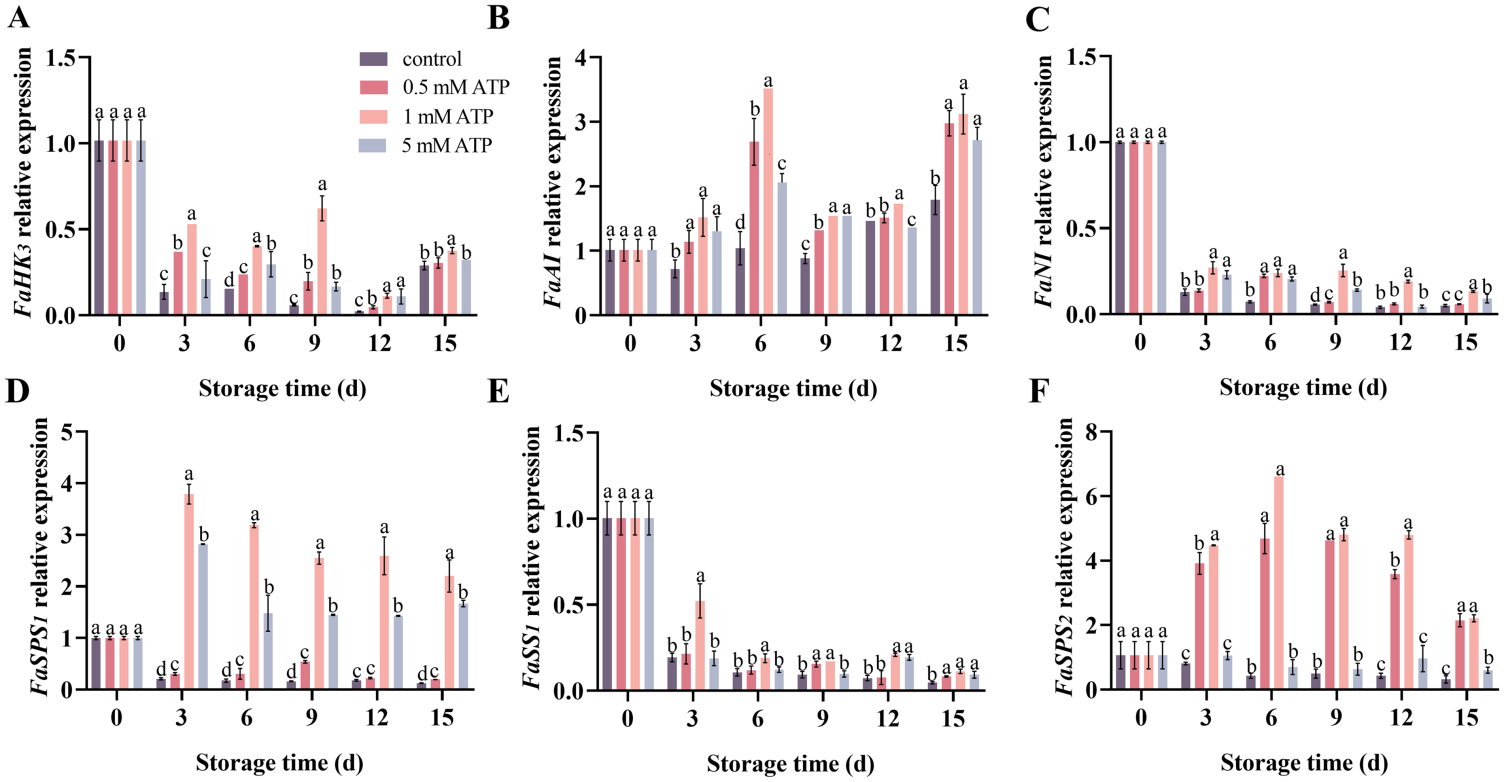
The related expression of sugar metabolism related genes in strawberries. **(A)** *FaHK3*, **(B)** *FaAI*, **(C)** *FaNI*, **(D)** *FaSS1*, **(E)** *FaSPS1*, and **(F)** *FaSPS2* relative expressions. Each value is the mean ± standard deviation from at least three strawberries. Different letters indicate significant differences among different treatments (*p* < 0.05).

As shown in Figure 6, the relative expression levels of sugar metabolism-related genes, including *FaHK3*, *FaNI*, *FaSS1*, *FaSPS1,* and *FaSPS2,* was decreased during storage time. The expression of *FaAI* exhibited a slight decrease during the storage period from days 0 to 9, followed by an increase from days 9 to 12. Compared with the control, ATP treatment significantly increased the relative expression of *FaHK3*, *FaAI*, *FaNI*, *FaSS1*, *FaSPS1*, and *FaSPS2*. Treatment with 1 mM ATP significantly increased the expression levels of *FaAI*, *FaSPS1,* and *FaSPS2* on days 3, 6, and 6, respectively.

### 3.7 Organic acid related genes involved in strawberries ripening

**Figure 7:**
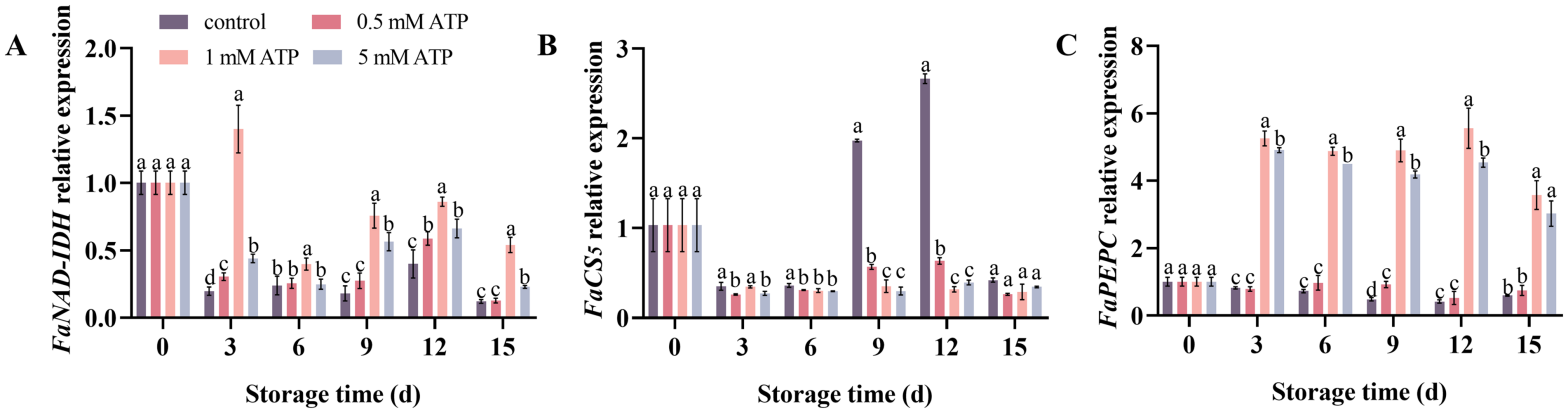
The related expression of organic acid related genes in strawberries. **(A)** *FaNAD-IDH*. **(B)** *FaCS5*. **(C)** *FaPEPC*. Each value is the mean ± standard deviation from at least three strawberries. Different letters indicate significant differences among different treatments (*p* < 0.05).

As strawberries ripened, the expression of *FaNAD-IDH* and *FaPEPC* decreased compared with those on day 0. The expression of *FaNAD-IDH* peaked on day 12 and then declined, with its level remaining lower than that on day 0 throughout the subsequent period. However, the expression of *FaPEPC* remained low throughout the storage period. ATP treatment significantly upregulated the expression of *FaNAD-IDH* and *FaPEPC*, with 1 mM ATP exerting the most prominent regulatory effect (Figures 7A,C). As strawberries ripened, the expression of *FaCS5* exhibited a trend of first increasing, peaking on day 12, and subsequently decreasing, whereas ATP treatment downregulated the expression (Figure 7B).

## 4 Discussion

ATP is an environmentally friendly and safe substance widely distributed in all organisms. As a novel post-harvest biological treatment strategy, ATP treatment has garnered extensive attention in academic and industrial fields. Exogenous ATP treatment plays a role in maintains the quality of fruits and vegetables during post-harvest storage (5, 32). In this study, exogenous ATP application markedly delayed the ripening process of strawberries, among which the 1 mM ATP treatment exhibited the most prominent regulatory effect on strawberry fruit ripening. Treatment with 1 mM ATP significantly delayed the color change and improved the firmness of strawberries. Consistently, Li et al. (33) found that ATP combined with CO_2_ treatment could significantly delay color change and improve the firmness of strawberry fruit, compared with those of the control.

At the initial stage of storage, organic substances in fruits can be converted into bioavailable nutrients, such as soluble sugars, amino acids, and vitamins, accompanied by a gradual increase in the TSS content. However, as the storage period increases, the decline in TSS in fruits can be attributed to the acceleration of respiration, and increased consumption of sugars and organic substances if there is a lack of sufficient organic material (34, 35). Our study indicated that ATP application after harvest could sustain TSS as well as sucrose, fructose, and glucose content. Relevant studies have revealed that ATP application can effectively slow the decline in post-harvest grape fruit firmness and TSS (16). ATP treatment can result in higher TSS levels in longan fruits, and reduce the fruit respiration rate, pulp breakdown index, and TA content (36). High CO_2_ levels reduce the content of fructose, glucose, and sucrose, whereas exogenous ATP delays the decline of these three sugars by maintaining the energy load and inhibiting fermentation metabolism (33). The accumulation of sugars in fruits and maintenance of quality are closely related to the activity of sugar-metabolizing enzymes (37).

Sugar serves as a plant carbon source and an essential substrate for energy supply, particularly sucrose, glucose, and fructose, which play critical roles in fruit quality (38–40). The sugar metabolic process is closely related to SS-s and invertase enzymes (41). AI, NI, and SS-lyase collectively participate in catalyzing the hydrolysis of sucrose, producing glucose and fructose. Conversely, sucrose synthesis is achieved through the synergistic action of SS-s and SPS, which convert glucose and fructose into sucrose (42, 43). SS-s is a glycosyltransferase with dual functions in synthesis and decomposition. Our results indicated that treatment with 1 mM ATP enhanced the activities of AI, NI, and SPS in strawberries, and inhibited the decline in SS-c, SS-s, and HK activities. The activities of the SS-s peaked on day 9 of storage, while HK, Ni and SS-C peaked on day 12 of storage. Similarly, the sucrose content decreased gradually after day 9 of storage, and treatment with 1 mM ATP delayed this process. This is consistent with the findings expressed by Duan et al. (7), which indicated that ATP treatment participates in the synthesis and decomposition of sucrose. Compared with the control, the 1 mM ATP treatment also significantly upregulated the relative expression levels of *FaHK3*, *FaAI*, *FaNI*, *FaSPS1*, *FaSPS2,* and *FaSS1*, which was consistent with the variation in enzyme activity. In addition, compared with the enhancement of synthase activity, the increase in invertase activity may be a key factor contributing to the decline in TSS during the later storage period. Therefore, it is speculated that ATP treatment could affect the enzyme activity by upregulated the expression of glucose metabolism related genes, thereby affecting the contents of sucrose, fructose and glucose, and delaying the reduction of TSS.

As the fruit ripened, organic acids gradually decreased because of the consumption of nutrients by respiratory metabolism. This study reveals that 1 mM ATP exerts a significant inhibitory effect on CS activity and gene expression levels in strawberries, and a positive correlation was observed between this inhibition and citric acid content in the fruits. CS is the key synthase affecting citric acid content in strawberries. Malic acid accumulation is negatively correlated with fruit NADP-ME and positively correlated with NADP-MDH activity (44). The activities of the NADP-MDH peaked on day 9 of storage, while NADP-ME peaked on day 12 of storage. The content of malic acid peaked at the same time as that of NADP-MDH, and treatment with 1 mM ATP delayed this process. Our study revealed that ATP treatment inhibited NADP-MDH activity and maintained higher NADP-ME activity. This indicated that ATP regulates the balance between malate biosynthesis and degradation by modulating the activities of these enzymes. PEPC catalyzes the carboxylation of phosphoenolpyruvate to generate OAA, which plays a critical role in the tricarboxylic acid cycle. ATP treatment can enhance the activity and gene expression levels of PEPC in fruits, which may be attributed to the substantial degradation of citrate, leading to the potential depletion of OAA. The upregulation of PEPC helps replenish OAA by fixing bicarbonate (HCO₃^−^), thereby maintaining post-harvest metabolic homeostasis in fruits. Furthermore, this study revealed a significant negative correlation between the citric acid content in fruits and expression of NAD-IDH. NAD-IDH is primarily located in the mitochondria and serves as the second control point of the tricarboxylic acid cycle. This enzyme catalyzes the conversion of isocitrate to *α*-ketoglutarate, which is one of the citrate degradation pathways (45). Inconclusion, it is speculated that ATP treatment could affect the enzyme activity by upregulated the expression of *FaPEPC* and *FaNAD-IDH*, while downregulating *FaCS5* expression, thereby affecting the contents of citric acid and malic acid, promoting the reduction of TA.

As a high-energy phosphate compound, ATP undergoes interconversion with adenosine diphosphate (ADP) in cells, which in turn enables the storage and release of energy. This process can provide energy for the normal life activities of cells and delay the ripening process of fruits and vegetables. Therefore, ATP plays a pivotal regulatory role in fruit ripening. Li et al. revealed that ATP treatment could maintain a high energy level, reduce damage of cell membrane integrity, inhibit the increase of respiratory rate, and ultimately delay the ripening of longan fruit (10). This study showed that ATP treatment, as a freshness maintenance method, could regulate the gene and enzyme activities of sugar and organic acid metabolism in fruits, affecting the contents of TSS and TA, increasing fruit firmness, thereby delaying fruit ripening. While in grape fruit, 1 mM ATP could effectively improve the firmness and TSS content of postharvest grapes, and increase the TA, effectively maintain grape quality (16). This may be due to the potential fruit-specific mechanisms. ATP is the key substance of energy metabolism and participates in multiple metabolic pathways. Therefore, it is necessary to further clarify the potential signaling pathways involved in exogenous ATP-mediated regulation of sugar and organic acid metabolism in strawberry fruits, which in turn affects fruit ripening.

In conclusion, through treatment with varying concentrations of ATP, it was found that 1 mM ATP could delay strawberry fruit ripening, improve color and fruit firmness, and reduce TA content while increasing TSS, thereby maintaining fruit quality. In sugar metabolism, 1 mM ATP treatment promoted the accumulation of glucose, fructose, and sucrose content in strawberries, and increased the activities of AI, NI, SS-s, SS-c, and HK, as well as the expression levels of *FaAI*, *FaSS1*, *FaSPS1*, *FaSPS2*, *FaNI*, and *FaHK3*. In organic acid metabolism, treatment with 1 mM ATP facilitated the degradation of citric and malic acid, elevated the enzymatic activities of PEPC and NADP-ME, and reduced the activities of NAD-MDH and CS enzymes. Concurrently, it upregulated the expression of *FaPEPC* and *FaNAD-IDH*, while downregulating *FaCS5* expression. This demonstrated that ATP treatment effectively promoted sugar accumulation and organic acid degradation.

## Data Availability

The raw data supporting the conclusions of this article will be made available by the authors, without undue reservation.

## Supplementary material

Supplementary Table 1. The primers of RT-qPCR.
